# Effects of the Chemokine Receptor 5 (CCR5)-Delta32 Mutation on Hepatitis C Virus-Specific Immune Responses and Liver Tissue Pathology in HCV Infected Patients

**DOI:** 10.5812/hepatmon.11283

**Published:** 2014-07-01

**Authors:** Abdulkerim Yilmaz, Hakan Alagozlu, Ozturk Ozdemir, Sema Arici

**Affiliations:** 1 Department of Gastroenterology, Cumhuriyet University, Sivas, Turkey; 2 Department of Medical Genetics, Cumhuriyet University, Sivas, Turkey; 3 Department of Medical Genetics, Canakkale Onsekiz Mart University, Canakkale, Turkey; 4 Department of Pathology, Cumhuriyet University, Sivas, Turkey

**Keywords:** Chemokines, Hepatitis C Virus, Infection

## Abstract

**Background:**

The specific antiviral T cells provide CC chemokine receptor 5 (CCR5) for the immune response during the hepatitis C virus (HCV) infection. Heterogenous and/or homozygous 32 base pair deletion in CCR5 gene (CCR5Δ32 bpdel) leads to reduced protein expression.

**Objectives:**

In the current case control study, we aimed to compare the histopathological findings of liver to the CCR5Δ32 bpdel mutation profiles, expression and some other clinical findings in patients with chronic HCV infection.

**Materials and Methods:**

Multiple Strip Assay reverse hybridisation and Real Time PCR techniques were used to determine the germline CCR5 mutations and immunohistochemical technique was used to evaluate the gene expression in targer tissue biopsies.

**Results:**

Target CCR5 WT/WT, WT/Δ32, and Δ32/Δ32 genotypes were observed in 91.4%, 8.6% and 0.0% for HCV positive patients and 98.3%, 1.7% and 0.0% for control group respectively. The histologic activity index (HAI) was significantly lower (4.0 ± 1.0) in the mutated group than the non-mutated group (5.7 ± 1.0). Decreased fibrosis levels were detected in HCV positive mutated group.

**Conclusions:**

Results showed that CCR5 polymorphism was more frequent in HCV positive patients than in healthy population in Turkish population. Current results also showed that mutated CCR5 signalling pathway due to CCR5-Delta32 may potentially result in subtle reduction of HCV specifity to the drug responses due to the positive impact on liver inflammation, fibrosis levels and liver destruction in HCV infection.

## 1. Backgraund

The chemokines are cytokines that stimulate the chemo taxis of the leukocytes and the stem cells in inflammation and homeostasis. CCR5 and CXCR3, which are T helper-associated (Th1) chemokine receptors are reported to contribute to hepatic inflammation in cases of hepatitis C virus (HCV) infection (1-4). The individuals who are homozygous for the 32-base-pair deletion (Δ32 bpdel/Δ32 bpdel) in the CCR5 gene are resistant to HIV-1 infection while heterozygote individuals are reported to have a slow course of the disease and this deletion is protective against the opportunist infections. Based on these trials, anti-chemokine and chemokine receptor blocker therapies have been introduced (5, 6). It was reported that CD3 positive T cells derived from the CCR5 heterozygote mutation decreased the CCR5 expression and the response to ligands in recent trials. The trials performed based on these findings reported that CCR5 mutation and expression could be involved in the modulation of the liver inflammation (7-9). Recently, it was well established that there was a correlation between the CCR5 mutation and the HCV infection. In these patients in whom mutation was detected, the liver inflammation and the grade of fibrosis were reported to be lower with a non-aggressive clinical course (10-15). 

## 2. Objectives

In the current case control study, it was aimed to find out the type and prevalence of the CCR5 mutation and its correlation to the hepatic expression on the histopathological liver findings in patients with chronic HCV.

## 3. Materials and Methods

### 3.1. Patients, Clinical Diagnosis and Laboratory Assessment

The current multidisciplinary study was performed at faculty of medicine of Cumhuriyet University between September 2008 and September 2009 by three disciplines; clinical diagnosis and therapy made by department of gastroenterology, genotyping was performed in department of medical genetics and histopathological tissue diagnosis and immunohistochemical analysis made by pathology. Study was approved by the Ethical Committee of the Cumhuriyet University Medical Faculty (approval no. 26/04/2009 09/58). In a total of 58 patients of chronic hepatitis C [(33 females (56.9%) and 25 males (43.1%)] were included in the current case control study. Results were compared to the control group that consisted of 58 healthy subjects [(29 females (34.5%) and 38 males (65.5%)].

The histopathological findings of liver and the function tests and the association with the virological parameters in control, mutated and non-mutated patients were compared. In addition, target CCR5 gene expression in the liver biopsies were examined for CHC patients and compared to the healty control and non-mutated patients histopathologically and immunohistochemically. Patients with a positive HCV antibody as determined by ELISA and a significant HCV RNA level (> 50 IU/mL) as determined by PCR were included in the trial. All patients were treatment-naive. The patients of seropositive for HAV, HBV, HDV, HIV, autoimmune hepatitis, alcohol history and toxic agent use were excluded from this study. The histologic activity indices (HAI) determined by the Modified Histological Activity Index (ISHAC) and fibrosis levels for both groups were compared.

### 3.2. Mutation Analysis

In a total of 116 (58 experimental and 58 individuals of control group) peripheral blood samples with EDTA were used for genomic DNA isolation in the current study. Total genomic DNA was extracted from peripheral blood samples from each individual by both automated Magna Pure Compact (Roche) and Invitek kit extraction techniques (Invitek^®^; Invisorb spin blood, Berlin, Germany) manually. Target CCR5 gene was genotyped by Strip Assay reverse hybridisation technique and confirmed by Real Time PCR, Light Cycler 2.0 methods (Roche) and for all patients. Target gene was simultaneously amplified in a biotin-labeled single multiplex amplification reaction (Viennalab^®^; PGX-HIV Strip Assay, Vienna, Austria) which is based on the reverse-hybridization principle automatically. The multiple polymerase chain reaction (PCR) was performed in a Perkin Elmer 9600 and the profile consisted of an initial melting step of 2 minutes at 94°C; followed by 35 cycles of 30 seconds at 94°C, 30 seconds at 61°C, and 30 seconds at 72°C; and a final elongation step of 7 minutes at 72°C. High portion of samples were also analysed by real-time PCR technique (LightCycler 2.0, Roche).

Briefly, Light Cycler Fast Start DNA Master HybProbes, master mix (water, PCR-grade, MgCl_2_, stock solution, Primer mix, HybProbe mix) and DNA template were used for real-time amplification. The multiple polymerase chain reaction (PCR) was performed for target polymorphic sequence in a Perkin Elmer 9600 and the protocol consisted of an initial melting step of 2 minutes at 94°C; followed by 35 cycles of 15 seconds at 94°C, 30 seconds at 58°C, and 30 seconds at 72°C; and a final elongation step of 3 minutes at 72°C. Software programme (LightCycler 2.0, Roche) was used for detection of the mutated and normal genotype profiles of target gene in the current control and HCV patients.

### 3.3. Histopathological and Immunohistochemical Methods

HE-stained liver preparations of 50 patients were re-examined and 3 to 5 µm thick sections were prepared from the paraffin blocks of these preparations on the adhesive-containing lames for immunohistocehmical staining. By administering the CCR5 (Goat Polyclonal anti-CCR5, NB100-714) stain to the cases immunohistochemically, the immunohistochemical expression of CCR5 in the liver sections with chronic hepatitis C infection was investigated. The avidin-biotin peroxidase (ABP) method was employed. The histopathological and immunohistochemical assessment showed CCR5 positivity in the portal region and the in the Kupffer cells in the parenchyma, and rare and weak positivity in the lymphocytes.

### 3.4. Statistical Analysis

In the current results the odds ratio and P Values were used to estimate the alleles frequency of CCR5 gene in HCV positive patients. Mutational variables were analyzed by using Fisher’s exact test. The software SPSS for Windows version 14.0 was used to perform statistical analysis. The Mann-Whitney U and chi-square tests were used to analyze differences between the patients and the controls. The estimate risk was examined by multivariate logistic regression analysis. Results were given as the mean (standard deviation [SD]) and the P Values less than 0.05 were considered as statistically significant.

## 4. Results

Demographic and laboratory characteristics of the patients were showed in Table 1. Fifty-eight patients with chronic hepatitis C and age-matched healthy controls were included in the current case-control study. Target CCR5 WT/WT, WT/Δ32, and Δ32/Δ32 genotypes were observed in 91.4%, 8.6%, and 0.0% for HCV positive patients and 98.3%, 1.7%, and 0.0% for control group respectively. Difference for heterozygous point mutation was not statistically significant (P = 0.206), (Table 2). No homozygous mutations (Δ32/Δ32) were detected in the presented cohort. While the viral load was 6.4 × 106 ± 2.9 × 105 IU/mL in the patient group with mutation, it was 2.8 × 106 ± 1.1 × 105 IU/mL in the group without mutation. The viral load from the patients with mutated CCR5 (WT/Δ32) was significantly higher when compared to the CCR5 (WT/WT) group (P = 0.037). HAI values for HCV positive mutated group (4.0 ± 1.0) were lower than the non-mutated group (5.7 ± 1.7) and the difference was statistically significant (P = 0.022). The ALT values were nearly the same for both groups (55.2 ± 32.4; 65.6 ± 38.5) and difference was not significant (P = 0.582). The fibrosis level was 1.6 ± 1.3 and 2.6 ± 1.8 in the group with and without mutation, respectively. The level of fibrosis was lower in the group with mutation; however the difference was not statistically significant (P = 0.352). Association between mutation, histopathological values and the distinct laboratory values of all studied groups were presented in Table 3. The HAI values were 6.1 ± 2.3 and 4.9 ± 1.3 in the group with and without expression, respectively. The difference was statistically significant (P = 0.034). The fibrosis level was 2.1 ± 2.1 and 1.9 ± 1.5 in the group with and without expression, respectively. The level of fibrosis was lower in the group without expression; however the difference was not statistically significant (P = 0.625), (Table 4). While 15 of the 45 patients without mutation among those with hepatitis C had tissue expression (no investigation could be performed in eight patients due to technical reasons), none of the five patients with mutation had expression. The difference was not statistically significant (P = 0.305) (Table 5).

**Table 1.** Demographic and Laboratory Characteristics of the Patients^a^

	WT/WT	WT/del
HCV patients		
Number	53	5
Sex, male/female	26/27	3/2
Age, y, Mean ± SD	54.4 ± 11.3	54.2 ± 7.8
**Genotypes**		
1A	3	-
1B	35	3
2A	3	-
Undetectable	12	2

^a^ Abbreviations: WT, wild; del, deletion; HCV, hepatitis C virus.

**Table 2.** Comparison of the Mutation Rate Between Patient and the Control Group^a^

CCR5 Gene Profiles	HCV	Control
**WT/WT**	53 (91.4)	57 (98.3)
**WT/∆32**	5 (8.6) ^b^	1 (1.7) ^b^
**∆32/∆32**	-	-
**Total**	58 (100)	58 (100)

^a^ Data are presented as Mean ± SD.

^b^ P = 0.206.

**Table 3.** The Comparison of the Histopathological and Laboratory Findings According Mutation^a,b^

HCV Patient Group	WT/WT	WT/∆32	P Value
**Viral load, IU/mL**	2.8 × 106 ± 1.1 × 105	6.4 × 106 ± 2.9 × 105	0.03^c^
**HAI**	5.7 ± 1.7	4.0 ± 1.0	0.022^c^
**Fibrosis**	2.6 ± 1.8	1.6 ± 1.3	0.352
**ALT**	65.6 ± 38.5	55.2 ± 32.4	0.582

^a^ Abbreviations: ALT, alanine transaminase; HAI, histology activity index; HCV, hepatitis C virus; WT, wild.

^b^ Data are presented as Mean ± SD.

^c^P < 0.05.

**Table 4.** The Comparison of Histopathological Findings According to the Intrahepatic Expression of CCR5^a^

	CCR5 Exp (+)	CCR5 Exp (-)	P Value
**Number**	15 (30)	30 (70)	
**HAI**	6.1 ± 2.3	4.9 ± 1.3	0.034^b^
**Fibrosis**	2.1 ± 2.1	1.9 ± 1.5	0.625

^a^ Abbreviations: HAI, histology activity index; Exp, expression.

^b^ P < 0.05.

**Table 5.** Mutation Type and Allele Frequency of CCR5-Delta32 Gene in Current Control Group and HCV Patients

Genotype	No.	Exp (+)	Exp (-)
**WT/WT**	45	15^a^	30
**WT/∆32**	5	0^a^	5
**Total**	50	15	35

^a^ P = 0.305.

## 5. Discussion

Acute and/or chronic HCV infection promotes a wide range of innate and adaptive immune responses in human. Permanent viral clearance occurs in a total of 20-50% of chronic HCV infected individuals. The incidence of CCR5 gene polymorphism is reported to be 10-15% in patients with chronic hepatitis C. It was similar to the rate of normal population (8, 10, 12). Our study confirmed previous data. Mutation rate was 8.6% in patients with chronic hepatitis C. However, we found lower mutation rates in healthy controls (1.7%) than reported by previous studies. We suggested that further studies required determining the association between treatment success and mutation incidence in patients with chronic HCV. Goulding et al. (12) reported a tendency for less severe hepatic inflammatory scores in chronic HCV patients with CCR5 delta heterozygote mutation and that this score was closely correlated with the mutation due to reduced migration of CCR5 expressing cells and thus a decreased hepatic inflammation. In the same trial, in the patients with heterozygote mutation, fibrosis level was increased and ALT levels were low; however the correlation of these two parameters with mutation was reported to be weak (12). Similar to previous studies, we found a positive effect of mutation on hepatic inflammation and fibrosis.

Wald et al. (10) reported that liver inflammatory activity was found to be significantly reduced (P = 0.005) in Jewish Israeli patients infected with the hepatitis C virus (HCV) carrying the CCR5 Delta32 allele. They suggested that CCR5 Delta32 allele may play a role in clinical course and the progression to the end stage in patients with chronic HCV. Mascheretti et al. (11) reported that the CCR2 mutation of the chemokine receptors could affect the viral clearance while the other CCR gene mutations could not affect the clinical course, the grade of fibrosis and the rate of treatment response in patients with chronic HCV. Our results were not supported to these findings. However, there were several trials supported that the role of chemokines. Chronic HCV is characterized by a nonspecific inflammatory infiltrate in the liver, mainly composed of CD8^+^ cells (3, 16). The interaction between the intrahepatic chemokines and its receptors is required for chemotaxis of the inflammatory cells. If there is a mutation in chemokine’s receptors, the interaction cannot occur. In conclusion, the course of the inflammation process seems to be slower. Woitas et al. (9) reported a high viral load in CCR5 mutation-carrier chronic HCV patients. We also detected a high viral load and low inflammation in patients with mutation. In our study, HAI was low in the group with mutation while the viral load was high in the same group. It is known that low HAI decreases the viral clearance, thereby increasing the viral load. This situation can be explained as an immune tolerance period.

We determined a significant correlation between the CCR5 mutation and the grade of hepatic inflammation and the viral load. We suggested that the mutation may decrease the inflammation, thus it can increase the viral load. The group without mutation was detected to have higher values for fibrosis and ALT than to the group with mutation; however the difference was not statistically significant. Also, we observed that mutation caused to decrease the fibrosis and ALT levels. The impact of the mutation on the inflammation also leads to a low ALT level. The same association could also be considered for the viral load.

Zhdanov et al. reported that the values for CC chemokines and the expression of the associated receptors in the liver tissue were higher compared to the blood values and that expression of CCR1 and CCR5 mRNA in blood was directly correlated with histological activity index and degree of fibrosis (14). Kusano et al. reported that the expression of the CC chemokines was increased in the portal and periportal regions and that this increase was correlated with inflammation (15). Similarly, it was reported that the increased tissue expression of the chemokines and the chemokine receptors was correlated with the tissue inflammation and the fibrosis level in patients with chronic HCV (17). Moreover, the chemokine and chemokine receptor polymorphism was decreased the tissue expression and that this association positively affected the tissue damage (17). Larrubia et al. reported a decreased chemokine and chemokine receptor level after anti-viral treatment in patients with chronic HCV (16). It was concluded that in the patients with chronic hepatitis C, there were increased levels of intrahepatic chemokine and chemokine receptors and it was correlated with the degree of hepatic inflammation (2, 13, 18). Success clearance of HCV require activated immuno-competent cells that is important for an effective immune response to HCV infection (19, 20). Chemokines and chemokine receptors have been shown to be critically involved in these processes (20) and CCR5Δ32 bpdel carriers of HCV positive patients (as presented in the current study) remain poor clearance during the HCV therapy by the masking of some other related genotypes (20, 21). Ahlenstiel et al. have claimed that point mutation in CCR5-Delta32 interrupts the CCR5 signalling pathway due to may potentially result in subtle reduction of HCV specific IFN-gamma responses in anti-HCV-positive haemophiliac patients (22). The current limited results and some other recent literature findings support the above hypotheses (23-26). CCR5 polymorphism is higher in HCV patients compared to the healthy population. We failed to detect receptor expression in the group of chronic HCV patients with mutation. Additionally, the HAI and fibrosis levels were also lower in these patients compared to the group without mutation. However, the group without mutation had a high level of expression and high levels of HAI and fibrosis. As a result, we concluded that the mutation decreased the expression, thereby leading to a reduction in the liver damage. As claimed by Rauch et al. the host genetic determinants such as; genetic polymorphisms of human leukocyte antigens, killer immunoglobulin-like receptors, chemokines, interleukins and interferon-stimulated genes are outcome and revealed the influence of hepatitis C clearance on spontaneous of hepatitis C infection (24). In our previous case-control study increased CCR2-V64I polymorphism was detected in chronic renal failure patients that requiring long-term hemodialysis when compare to the healthy controls (27). Human monoclonal antibodies against CCR5, CXCR3 and their ligands have been used to treat different inflammatory and infectious diseases in humans and in animal models such as HIV-1 (16). In chronic HCV infection, the chemokines may cause a progression in the liver injury. In the future, chemokine receptor blockage and/or modulation may gain value as a successful treatment marker due to the fact that they decrease the inflammation. Further multicenter trials may be useful for identification the CCR5 on chronic HCV progression and data to be obtained from new trials may suggest that the chemokines and their receptors may be potential therapeutically targets in chronic HCV infection.

In conclusion, the liver tissue pathology and some other parameters such as; fibrosis, HAI, HCV polymorphism are important determinants for clearance outcome in HCV infection. The current results showed the possible role of polymorphic genes that encode chemokines in the spontaneous clearance or persistence of chronic HCV infection in Turkish population. Results should be supported by large prospective population-based studies.
